# Large, rapidly evolving gene families are at the forefront of host–parasite interactions in *Apicomplexa*

**DOI:** 10.1017/S0031182014001528

**Published:** 2014-09-26

**Authors:** ADAM J REID

**Affiliations:** Wellcome Trust Sanger Institute, Genome Campus, Hinxton, Cambridgeshire CB10 1SA, UK

**Keywords:** gene families, *Apicomplexa*, host–parasite interactions, antigenic variation, immune evasion, *Plasmodium*, *Toxoplasma*, *Eimeria*, *Babesia*, *Theileria*

## Abstract

The *Apicomplexa* is a phylum of parasitic protozoa, which includes the malaria parasite *Plasmodium*, amongst other species that can devastate human and animal health. The past decade has seen the release of genome sequences for many of the most important apicomplexan species, providing an excellent basis for improving our understanding of their biology. One of the key features of each genome is a unique set of large, variant gene families. Although closely related species share the same families, even different types of malaria parasite have distinct families. In some species they tend to be found at the ends of chromosomes, which may facilitate aspects of gene expression regulation and generation of sequence diversity. In others they are scattered apparently randomly across chromosomes. For some families there is evidence they are involved in antigenic variation, immune regulation and immune evasion. For others there are no known functions. Even where function is unknown these families are most often predicted to be exposed to the host, contain much sequence diversity and evolve rapidly. Based on these properties it is clear that they are at the forefront of host–parasite interactions. In this review I compare and contrast the genomic context, gene structure, gene expression, protein localization and function of these families across different species.

## INTRODUCTION

Apicomplexans are parasitic protozoa, which possess an apical complex, a unique set of organelles involved in invasion of host cells. The phylum includes the malaria parasite genus *Plasmodium, Toxoplasma gondii* – a parasite capable of infecting virtually all cell types in all warm-blooded animals – and a plethora of other parasites of humans and livestock: *Cryptosporidium, Eimeria, Neospora, Theileria* and *Babesia*. Thanks to improving technologies, large financial investment and much hard work over the past decade we have been furnished with annotated genome assemblies for the majority of important apicomplexans. Many of their genes are conserved across the phylum, representing the core genome of an apicomplexan parasite. However each genome has revealed unique genes and gene families, which should to tell us about the environmental niche of each parasite and its survival strategy. A gene family is a set of genes with similar sequences, domain structure and function that are thought to have a common ancestor.

By infecting the bodies and invading the cells of complex organisms, apicomplexan parasites endanger the life of their host and open themselves to attack from its immune system (Turner, 2002 #515). To be successful the parasite must optimize its chances of passing into a new host. This requires the parasite to minimize damage to its host until it finds a new host, while preventing the immune system from killing it. Good strategies for achieving these goals will differ in different tissues and in different hosts. Thus, in each parasite, specialized genes should have evolved to satisfy these needs. Evidence points to the large, rapidly mutating, clade-specific gene families having a large role to play in these processes.

These gene families are sometimes referred to as contingency gene families. Contingency genes have been defined as those which allow enhanced phenotypic variation (Bayliss *et al.* 2001). These genes are found in large families and are thought to be subject to higher mutation rates than other genes, by processes such as gene conversion, recombination, duplication and deletion. They often reside in specialized regions of the genome, which may facilitate mutation and correct gene expression. Many examples encode surface antigens: membrane-bound proteins to which the host immune system develops antibodies. The classical example in Apicomplexa is the *var* family from *Plasmodium falciparum*, which is involved in antigenic variation (Guizetti and Scherf, 2013). However, other families encode products which are secreted into the host cell. These perform roles such as remodelling the host cell to favour the parasite (e.g. ROP kinases of the *Coccidia*, FIKK kinases in *P. falciparum* and SVSPs in *Theileria* spp.). Each new group of apicomplexan parasites investigated reveals several unique multi-gene families. In this review I will discuss these large gene families in apicomplexan parasites: how genome sequencing has been used to reveal their full extent in each genome, their genomic context, functions and how those functions relate to different parasitic niches.

## IDENTIFYING GENE REPERTOIRES

Whole genome sequencing has dramatically improved our understanding of large variant gene families, their extent, diversity and genomic context. However, it has remained challenging to accurately determine their full repertoires. While contingency gene families make it difficult for the human immune system to battle parasites, they also make it difficult for us to produce complete genome assemblies, frustrating our attempts to better understand them. This is because these families are inherently repetitive. The whole or part of one gene may look much like that of another gene. This is likely key to their functional role, but also makes it difficult to piece together the genome.

The *var* gene family of *P. falciparum*, involved in antigenic variation and sequestration, is a prime example. Only using a variety of genome sequencing technologies, as well as manual finishing work, over several years have we become fairly sure that the *P. falciparum* 3D7 reference genome sequence is complete and describes the full complement of *var* genes (M. Berriman, personal communication). The problem is that each new parasite strain sequenced has a largely distinct set of *var* genes, variously recombined and mutated. This makes it difficult to determine the set of *var* genes in each new strain using current technologies without laboriously generating a completely new genome assembly. Furthermore, repetitive parts of these genes are sometimes longer than sequencing reads, meaning that de novo assembly approaches will fail to resolve them. Instead, researchers have been exploring targeted *de novo* assembly strategies to explore *var* gene repertoires in worldwide strains of *P. falciparum* (Assefa, 2013). Similarly, it was found that the *T. gondii* genome sequence contained collapsed repeats of the *rop5* gene, a member of the *ropk* family (Reese *et al.* 2011). A genetic linkage analysis had identified the locus of this gene as important for parasite virulence. Detailed analysis of the locus identified the collapsed repeat, which was found to vary in copy number between parasites with differing virulence. Without continued improvement of genome sequences, using a variety of technologies, it is likely that key features of parasite biology will be missed.

Although it can be difficult to define every gene in a repetitive gene family, straightforward analysis of even a draft assembly will highlight much of their repertoire. Each new genome assembly of sufficient phylogenetic distance from those we have will likely identify new such families. Furthermore, sequencing more members of well-studied genera will be useful in understanding the evolution of these families. Table 1 summarizes our knowledge of these large families (those with greater than 20 members) in apicomplexan species sequenced to date.

**Table 1.** Contingency gene family repertoires of the A*picomplexa*

Family	Subfamily	Species	Number per genome	Genomic location	Gene expression	Cellular localization of product	Function(s)
*var*		*Plasmodium falciparum*	~60 (Gardner *et al.* 2002)	Subtelomeric/intrachromosomal (Gardner *et al.* 2002)	Single transcript variant in cloned parasites, exc. *var1csa* (Kyes *et al.* 2007). Single protein expressed on surface (Chen *et al.* 1998; Scherf *et al.* 1998)	MC, RBC surface (Kyes *et al.* 2001)	Cytoadherence and antigenic variation (Kyes *et al.* 2001), immunomodulation (D'Ombrain *et al.* 2007)
*SICAvar*		*Plasmodium knowlesi*	192 (Pain *et al.* 2008)	Not specific (Pain *et al.* 2008)	Multiple (protein) (Howard *et al.* 1983)	RBC surface (Howard *et al.* 1983)	Antigenic variation, virulence (Barnwell *et al.* 1983)
		*Plasmodium cynomolgi*	2 (Tachibana *et al.* 2012)	Not specific (Tachibana *et al.* 2012)	–	–	–
*Pv-fam-a (PvTRAG)*		*Plasmodium vivax*	36 (Tachibana *et al.* 2012)	Subtelomeric (Carlton *et al.* 2008)	–	–	–
		*Plasmodium knowlesi*	26 (Tachibana *et al.* 2012)	Subtelomeric (Pain *et al.* 2008)	–	–	–
		*Plasmodium cynomolgi*	36 (Tachibana *et al.* 2012)	Subtelomeric (Tachibana *et al.* 2012)	–	–	–
*Pv-fam-e (RAD)*		*Plasmodium vivax*	44 (Tachibana *et al.* 2012)	Mostly internal arrays on chromosome 5 (Carlton *et al.* 2008)	–	–	–
		*Plasmodium cynomolgi*	27 (Tachibana *et al.* 2012)	Syntenic with *P. vivax* members (Tachibana *et al.* 2012)	–	–	–
*rif/stevor /pir*	*rif*	*Plasmodium falciparum*	163 (Logan-Klumpler *et al.* 2012)	Subtelomeric (Gardner *et al.* 2002)	Multiple variants expressed in asexual *in vitro* parasite population (Bozdech *et al.* 2003)	MC, PV, RBC surface (Kyes *et al.* 1999), A-type RBC (Khattab and Klinkert, 2006), A-type merozoite apex, B-type PV (Petter *et al.* 2007)	–
	*stevor*	*Plasmodium falciparum*	30 (Logan-Klumpler *et al.* 2012)	Subtelomeric (Cheng *et al.* 1998)	Two or three variants per cell in trophozoites (Kaviratne *et al.* 2002)	MC, PV, RBC surface (Niang *et al.* 2009), merozoite apex (ref)	–
	*pir*	*Plasmodium berghei*	100 (Otto *et al.*)	Subtelomeric (Hall et al. 2005)	–	RBC surface (Di Girolamo *et al.* 2008)	–
		*Plasmodium yoelii*	583 (Otto *et al.*)	Subtelomeric (Carlton *et al.* 2002)	1–3 variants per cell (Cunningham *et al.* 2009)	RBC surface (Cunningham *et al.* 2005)	–
		*Plasmodium chabaudi*	194 (Otto *et al.*)	Subtelomeric (Hall *et al*. 2005)	Multiple expressed in population (Lawton *et al.* 2012)	RBC surface (Janssen *et al.* 2004)	Putative role in virulence (Spence *et al.* 2013)
		*Plasmodium vivax*	346 (Carlton *et al.* 2008)	Subtelomeric (del Portillo *et al.* 2001)	Multiple variants per cell (Fernandez-Becerra *et al.* 2005)	RBC surface (del Portillo *et al.* 2001)	–
		*Plasmodium knowlesi*	67 (Pain *et al.* 2008)	Not specific	–	–	Putative role in molecular mimicry (Pain *et al.* 2008)
		*Plasmodium cynomolgi*	265 (Tachibana *et al.* 2012)	*Vir*-like (254) subtelomeric, *kir*-like (11) internal (Tachibana *et al.* 2012)	–	–	–
*Phist*		*Plasmodium falciparum*	71 (Sargeant *et al.* 2006)	Subtelomeric (Sargeant *et al.* 2006)	–	–	Modulation of erythrocyte cytoskeleton (RESA) (Pei *et al.* 2007)
		*Plasmodium vivax*	39 (Sargeant *et al.* 2006)	Subtelomeric (Sargeant *et al.* 2006)	–	–	–
		*Plasmodium cynomolgi*	21 (Tachibana *et al.* 2012)	Subtelomeric (Tachibana *et al.* 2012)	–	–	–
*fikk*		*Plasmodium falciparum*	20 (Schneider and Mercereau-Puijalon, 2005)	Subtelomeric (Schneider and Mercereau-Puijalon, 2005)	Multiple protein variants in blood stage (Nunes *et al.* 2007)	Inner surface of RBC membrane or within the parasite (Nunes *et al.* 2007)	Phosphorylation of host membrane skeleton proteins altering RBC mechanical properties (Nunes *et al.* 2010)
*RMP-fam-a (pyst-a)*		*Plasmodium berghei*	23 (Otto *et al.*)	Subtelomeric (Hall *et al*. 2005)	–	–	–
		*Plasmodium yoelii*	94 (Otto *et al.*)	Subtelomeric (Carlton *et al.* 2002)	–	–	–
		*Plasmodium chabaudi*	132 (Otto *et al.*)	Subtelomeric, one internal cluster (chr. 13) (Otto *et al.*)	–	–	–
*RMP-fam-b (pyst-b)*		*Plasmodium berghei*	34 (Otto *et al.*)	Subtelomeric (Hall et al. 2005)	–	–	–
		*Plasmodium yoelii*	48 (Otto *et al.*)	Subtelomeric (Carlton *et al.* 2002)	–	–	–
		*Plasmodium chabaudi*	26 (Otto *et al.*)	Subtelomeric (Hall et al. 2005)	–	–	–
*RMP-fam-c*		*Plasmodium yoelii*	22 (Otto *et al.*)	Subtelomeric (Carlton *et al.* 2002)	–	–	–
*srs*		*Toxoplasma gondii*	104 (Reid *et al.* 2012)	Intrachromosomal, in tandem arrays of various size (Reid *et al.* 2012)	Multiple variants in tachyzoites (Tomavo, 1996)	Parasite surface (Tomavo, 1996)	Cytoadherence (prior to invasion) (Jung *et al.* 2004), immune evasion (Kim and Boothroyd, 2005).
		*Neospora caninum*	227 (Reid *et al.* 2012)	Intrachromosomal, in tandem arrays of various size (Reid *et al.* 2012)	Multiple variants in tachyzoites (Reid *et al.* 2012)	Parasite surface (Howe *et al.* 1998)	–
*susa*		*Toxoplasma gondii*	26 (Reid *et al.* 2012)	Intrachromosomal, with three tandem arrays (Reid *et al.* 2012)	Multiple variants in tachyzoites (Reid *et al.* 2012). Expressed in bradyzoites (Pollard *et al.* 2008)	Parasite surface (Pollard *et al.* 2008)	–
		*Neospora caninum*	38 (Reid *et al.* 2012)	Intrachromosomal, with three tandem arrays (Reid *et al.* 2012)	None in tachyzoites (Reid *et al.* 2012)	–	–
*sag*		*Eimeria* spp.	16–172 (Reid, 2014 #517)	Intrachromosomal, with few, large tandem arrays (Reid, 2014 #517)	Multiple variants (Tabares *et al.* 2004)	Parasite surface (Tabares *et al.* 2004)	–
*esf1*		*Eimeria spp.*	18–50 (Reid, 2014 #517)	Maybe in tandem (Reid, 2014 #517)	Multiple variants across different life stages (Reid, 2014 #517)	–	–
*esf2*		*Eimeria spp.*	2–23 (Reid, 2014 #517)	Tandemly arrayed (Reid, 2014 #517)	Multiple variants across different life stages (Reid, 2014 #517)	–	–
*ropk*		*Toxoplasma gondii*	55 (Talevich and Kannan, 2013)	Intrachromosomal, some small arrays (Reese *et al.* 2011)	Multiple variants in tachyzoites (Reid *et al.* 2012)	ROP18 localizes to host cytosol/PVM (Taylor *et al.* 2006), ROP16 localizes to host nucleus (Saeij *et al.* 2007)	ROP18 protects PVM from attack by host IRGs (Fentress *et al.* 2010)., ROP16 modifies host cell signalling (Saeij *et al.* 2007).
		*Neospora caninum*	44 (Talevich and Kannan, 2013)	Intrachromosomal (Reid *et al.* 2012)	Multiple variants in tachyzoites (Reid *et al.* 2012)	–	–
		*Eimeria* spp.	28 (Talevich and Kannan, 2013)	Intrachromosomal (Reid, 2014 #517)	Multiple variants across different life stages (Reid, 2014 #517)	–	–
*ves*	*ves1*	*Babesia bovis*	72 *ves1alpha* , 43 *ves1beta* (Brayton *et al.* 2007)	Intrachromosomal, mostly arrayed in ves1alpha/beta pairs (Brayton *et al.* 2007)	Single transcript transcribed (Zupanska, 2009 #516)	RBC surface (O'Connor *et al.* 1997)	Cytoadherence (O'Connor and Allred, 2000), antigenic variation (Allred *et al.* 2000)
		*Babesia bigemina*	74 *ves1a*, 80 *ves1b* (Jackson *et al.* 2014)	–	–	–	–
		*Babesia divergens*	202 *ves1* (Jackson *et al.* 2014)	Subtelomeric tandem arrays (Jackson *et al.* 2014)	–	–	–
	*ves2*	*Babesia bigemina*	116 (Jackson *et al.* 2014)	–	–	Secreted? (Jackson *et al.* 2014)	–
		*Babesia divergens*	95 *ves2a*, 62 *ves2b* (Jackson *et al.* 2014)	–	–	Secreted? (Jackson *et al.* 2014)	–
*smORF*		*Babesia bovis*	44 (Brayton et al, 2007)	Intrachromosomal, associated with *ves1* genes (Brayton *et al.* 2007)	Multiple variants transcribed (Brayton *et al.* 2007)	Secreted? (Jackson *et al.* 2014)	Analogous to ves2? (Jackson *et al.* 2014)
*bmn*		*Babesia microti*	24 (Cornillot *et al.* 2012)	Subtelomeric and intrachromosomal (Cornillot *et al.* 2012)	Single variant protein (Homer *et al.* 2000)	–	–
Tpr/Tar		*Theileria parva*	39 (Gardner *et al.* 2005)	Single tandem array of 28, 11 dispersed copies (Gardner *et al.* 2005)	–	Predicted integral membrane proteins (Weir *et al.* 2010)	–
		*Theileria annulata*	93 (Weir *et al.* 2010)	Dispersed in small arrays (Pain *et al.* 2005)	–	Predicted integral membrane proteins (Weir *et al.* 2010)	–
*svsp*		*Theileria parva*	85 (Gardner *et al.* 2005)	Subtelomeric tandem arrays (Gardner *et al.* 2005)	All genes expressed in macroschizont (Schmuckli-Maurer *et al.* 2009)	Predicted secreted into host cell (Weir *et al.* 2010)	–
		*Theileria annulata*	51 (Weir *et al.* 2010)	Subtelomeric tandem arrays (Pain *et al.* 2005)	All genes expressed in macroschizont (Pain *et al.* 2005)	Predicted secreted into host cell (Weir *et al.* 2010)	–
SfiI subtelomeric		*Theileria parva*	50 (Gardner *et al.* 2005)	Subtelomeric (Gardner *et al.* 2005)	–	–	–
		*Theileria annulata*	72 (Weir *et al.* 2010)	Subtelomeric (Pain *et al.* 2005)	–	–	–
Mucin-like glycoproteins		*Cryptosporidium parvum*	20–30 (Abrahamsen *et al.* 2004)	–	–	Parasite surface (Chatterjee *et al.* 2010)	Tethering of sporozoite to oocyst wall (Chatterjee *et al.* 2010)

Here we show repertoires of large gene families (>20 members) for species with published genome sequences. Additional information on expression, localization and function is included where available. Some families may be missing in some species where they have not been described in the accompanying publication. Abbreviations: RBC, red blood cell; MC, Maurer's cleft; PV, parasitophorus vacuole.

## GENOMIC CONTEXT

**Fig. 1.**
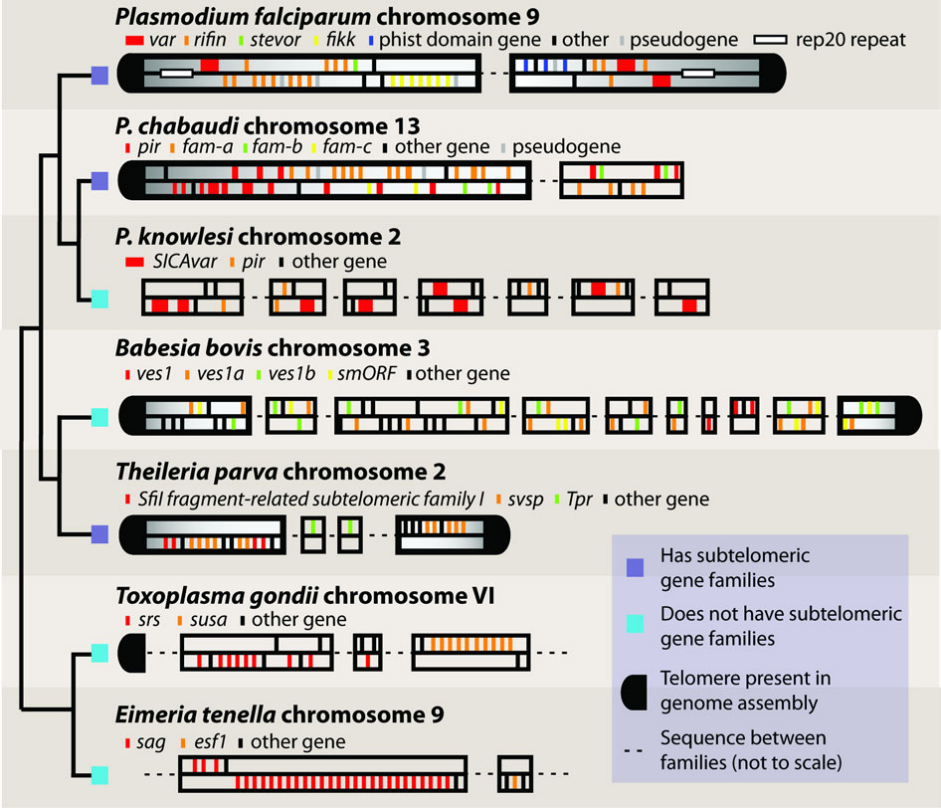
Genomic context of large gene families in Apicomplexa. Gene families from several species are shown in their genomic context using example chromosomes. Only those gene families described in Table 1 are shown, with dashed lines representing gaps in between. The figure is not to scale. Genes are shown on their coding strand. Colours are specific to each species and are not meant to imply any homology between families in different species, even where this exists. Telomeric sequences are highlighted where they are present in the genomic assemblies. Subtelomeres are highlighted where genes families proximal to them are those described in Table 1. A cladogram shows the known relationships between species and highlights those, which specifically organize their gene families at telomeres and those, which do not. Genome sequences were downloaded from either GeneDB (Logan-Klumpler *et al.* 2012) or EuPathDB (Aurrecoechea *et al.* 2009): *P. falciparum* (Gardner *et al.* 2002), *Plasmodium chabaudi* (Otto *et al.*), *P. knowlesi* (Pain *et al.* 2008), *B. bovis* (Brayton *et al.* 2007), *T. parva* (Gardner *et al.* 2005), *T. gondii* (Gajria *et al.* 2008), *Eimeria tenella* (Reid, 2014 #517).

Complete genome sequences have allowed us to analyse the genomic context of gene families in unprecedented detail. Genomic context appears to be important for regulation of gene expression and generation of diversity in large apicomplexan gene families. In *P. falciparum* the *var, rif* and *stevor* families cluster together, proximal to each telomere (Fig. 1). The *phist* and *fikk* families are also found in these subtelomeric regions but further towards the centromeres.

Subtelomeric location is common to contingency families in almost all species of *Plasmodium* examined. It has been suggested to play a role in regulating expression and generating diversity by promoting recombination (Scherf *et al.* 2008). The occasionally zoonotic monkey malaria parasite *Plasmodium knowlesi* is an exception. In *P. knowlesi*, both *SICAvars* and *pirs* are spread throughout the chromosomes (Fig. 1). However, despite their internal location, they are associated with telomere-like repeats, which might play a role in promoting recombination (Pain *et al.* 2008). *Var* genes (excluding the highly conserved *var1csa* and *var2csa*) can be classified into three types based on their promoter sequences: *upsA, upsB* and *upsC* (Lavstsen *et al.* 2003). These distinct promoters are associated with distinct contexts. *UpsB var* genes are subtelomeric and transcribed away from the telomere, *upsA* are subtelomeric, but transcribed towards the telomere. *UpsC var* genes are found in core chromosomal regions. The telomeres of *P. falciparum* chromosomes (Freitas-Junior *et al.* 2000) and also internal *var* genes (Ralph *et al.* 2005) have been shown to localize in a small number of foci at the periphery of the nucleus. This also occurs in rodent malaria parasites and is thought to foster recombination, generating new variants (Figueiredo *et al.* 2000).

Subtelomeric gene families are also a feature of *Theileria* spp. but not of *Babesia* or any *Coccidia* so far examined (Fig. 1). A much greater sampling of the *Apicomplexa* will be required to understand just how common this arrangement is and whether it has evolved multiple times in *Apicomplexa*.

Relative orientation of genes is important for expression of the *ves1* gene family in *Babesia bovis. Ves1/smORF* units comprise a handful of genes, generally with at least one *ves1a*, one *ves1b* and a *smORF* (Brayton *et al.* 2007). *Ves1α* and *ves1β* together encode VESA proteins, involved in antigenic variation and cytoadherence (Allred *et al.* 2000; O'Connor and Allred, 2000). The function of *smORF* is not known. The *ves1* family is one of two known amongst apicomplexans, which shows true properties of antigenic variation, with only one variant expressed at a time. Transcription from the functional locus of active transcription (LAT) is thought to require a *ves1α* and a *ves1β* in divergent orientation, separated by a bidirectional promoter (Al-Khedery and Allred, 2006). *Ves1*/*smORF* units are found scattered throughout *Babesia* chromosomes, not in subtelomeres or in tandem arrays (Fig. 1).

The *srs* and *sag* genes families, which encode unrelated, but structurally similar, surface antigens in *Coccidia* are found spread throughout chromosomes, mostly in tandem arrays (Fig. 1b). These arrays tend to contain genes, which are more similar to each other than other clusters, and tend to share domain subfamily architectures (Reid *et al.* 2012; Wasmuth *et al.* 2012). This is probably maintained through local gene duplication as well as gene conversion (Reid *et al.* 2012).

Tandem arrays of genes can easily arise through gene duplication but the maintenance of this pattern may have advantages for promoting gene conversion. In some cases genes within a tandem array may be more similar to each other than those in other arrays (e.g. *Coccidia srs*/*sag*). In other cases they are not e.g. *Plasmodium pirs* (Otto *et al.*) and *Theileria* *SfiI sub-telomeric* *genes* *(*Weir *et al.* 2010*)*, suggesting recombination between arrays. Similarity within loci might be due to either recent divergence, through copying or a continued process of local gene conversion. Some genes families, present in tandem arrays, may preserve their orientation, all being transcribed towards centromere or telomere, in order that they can line up with homologous arrays from other chromosomes or other parts of the same chromosome for recombination.

Currently it is clear that subtelomere context is not a common feature of large apicomplexan gene families. Indeed there is no evidence for it in *Coccidia*. In the haemosporidia (*Plasmodium*) and piroplasms (*Babesia, Theileria*) it may have been lost or gained multiple times.

## PROTEIN DOMAIN STRUCTURE

The most common feature of large apicomplexan gene family sequences is a signal peptide: a hydrophobic N-terminal sequence of 10–20 amino acids. This is an indication that such genes may be directly involved in host–parasite interactions as they can be exported from the parasite cell via the secretory pathway. Many of these gene families appear to be ultimately bound to membrane, using either transmembrane domains or glycophosphatidylinositol (GPI) anchors. In general, extracellular proteins tend to be rich in disulphide bonds to improve stability, and indeed apicomplexans are no exception (Fass, 2012). Despite these similarities, gene families in different groups of parasites share no detectable homology and in some cases can be shown to derive from different ancestral families.

The *var* gene family exhibits a complex protein domain architecture. *Plasmodium falciparum var* genes are composed of Duffy binding-like (DBL), CIDR and C2 domains (Gardner *et al.* 2002). DBL domains have six subfamilies and CIDR two. There is a great diversity of domain architectures in the *var* family, although the N-termini are usually composed of a DBL*α* domain followed by a CIDR domain, suggesting this structure is key to their function (Gardner *et al.* 2002). DBL domains are also found in several other genes of *P. falciparum*, which are involved in binding host cells and are required for invasion by the merozoite, implying an ancestral role for this domain in host cell binding (Tolia *et al.* 2005). The C-terminus includes a transmembrane domain, which tethers the proteins to the erythrocyte surface.

Other protein families have much simpler architectures. *Plasmodium falciparum rifs* have an N-terminal signal peptide, a PEXEL motif and either one or two transmembrane domains depending on the subfamily (Bultrini *et al.* 2009). PEXEL motifs are required for export of proteins to the red blood cell surface (Hiller *et al.* 2004; Marti *et al.* 2004). Based on remote sequence similarity, conservation of intronic sequence motifs and predicted protein secondary structure, it has been proposed that *rif* genes are related to the *pirs*, a family present in all other species so far sequenced (Janssen *et al.* 2004). These families are an example of those which have diverged almost beyond recognition, and which may help to provide insights into evolution of gene families in malaria.

Coccidian surface antigen gene families also use relatively simple domain structures. *Toxoplasma srs* genes usually contain two SAG domains (Jung *et al.* 2004), while the short, consistent lengths of *Eimeria sag* genes suggest a single domain structure (Reid, 2014 #517). Both families have signal peptides and GPI anchor addition sites. Furthermore, the individual domain sequences in each family both tend to contain six cysteines and are of similar lengths (200–300 amino acids). However, these common properties appear to be convergent, as the families have distinct origins. While *Toxoplasma srs* genes have been proposed to derive from metazoan ephrins by lateral transfer (Arredondo *et al.* 2012). *Eimeria sag* genes meanwhile are thought to be related to CAP domains (Reid, 2014 #517). CAP domains are widely used across the tree of life.

*Theileria* parasites invade and transform host leucocytes (reviewed in Dobbelaere and Kuenzi, 2004). It is thought that they transform the host cell using families of secreted proteins. On such example is the *SVSP* family, which encode a signal peptide and a *Frequently Associated IN Theileria* (FAINT) domain. FAINT domains are common to a range of *Theileria* subtelomeric genes from different families, all predicted to be secreted (Pain *et al.* 2005). It has been speculated that this low-complexity glutamine- and proline-rich domain may be difficult for vertebrate immune systems to recognize and therefore could provide some anonymity for the parasite (Gardner *et al.* 2005).

Domain structures in these families suggest a diverse array of structures and of phylogenetic origins for surface proteins and other families. This is likely to be a result of the requirement for constant innovation in the parasites’ battle with the host but also of differing requirements related to the niche of the parasite and its strategy for transmission.

## EXPRESSION PATTERNS AND PROTEIN LOCALIZATION

**Fig. 2.**
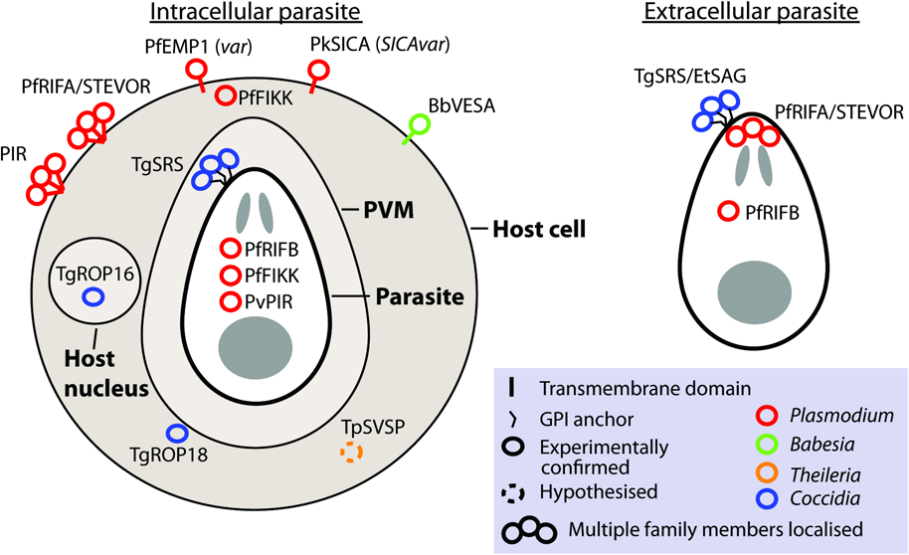
Localization of gene family products. The localization of gene products is shown for intracellular and extracellular parasites, where known or hypothesized. Where multiple copies have been localized this is indicated by a bundle of circles. Abbreviations: PVM, parasitophorus vacuole membrane. n.b. The PVM is not present in *Theileria* infected leucocytes and is destroyed quickly after invasion by Babesia (Asada, 2012 #518). Evidence: TgROP18 localizes to host cytosol/PVM (Taylor *et al.* 2006), TgROP16 localizes to host nucleus (Saeij *et al.* 2007), TpSVSP predicted to localize to host cytosol (Weir *et al.* 2010), PfRIF/STEVOR localize to parasite apex (Petter *et al.* 2007; Blythe *et al.* 2008) and RBC surface (Kyes *et al.* 1999), PvVIR14 and PvVIR10 are exported to the membrane of iRBC whereas PvVIR17 remains inside the parasite (Bernabeu *et al.* 2012), rodent PIR family localize on or close to the surface of the RBC (Cunningham *et al.* 2009), TgSRS multiple gene products have been localized to the parasite surface (Tomavo, 1996), EtSAG multiple gene products localized to the parasite surface (Tabares *et al.* 2004), PfFIKKs localize to Maurer's cleft/host membrane cytoskeleton and within parasite (Nunes *et al.* 2010), PfEMP1 (*var* family) localizes to the host cell surface (Kyes *et al.* 2001), PkSICA (SICAvar family) localizes to the host cell surface (Howard *et al.* 1983), BbVESA (ves1 family) localizes to the host cell surface (O'Connor *et al.* 1997).

Gene families involved in antigenic variation *sensu stricto* are expressed monoallelically, with only one family member localized to the parasite or host cell surface at a time (Borst, 1991). Although this is observed for *var* and *VESA* families, it is not what has been observed for most apicomplexan large gene families. Frequently, where measurements have been made, multiple members are expressed. Unlike switching of *vsg* in trypanosomes, switching to different genes in apicomplexans does not involve genomic rearrangement. Although many contingency gene families encode protein localized to the parasite or host cell surface, some families localize to the host cytosol or nucleus (e.g. *ropk*) and some families have different members with distinct localizations (e.g. *rif*; Fig. 2).

Correct expression of *P. falciparum var* genes involves the suppression of all but one gene and switching between individual genes; control occurs at the level of transcription initiation (Scherf *et al.* 2008). Expression is the highest early after cell invasion, presumably because the protein must quickly be expressed on the surface of the invaded red cell. Control of transcription is thought to require genetic elements such as the intron (Deitsch *et al.* 2001) and the *ups* promoter region (Voss *et al.* 2006). An ApiAP2 transcription factor is known to bind to the intron, which acts as a bidirectional promoter and from which non-coding RNA is transcribed (Zhang *et al.* 2011). *Plasmodium falciparum* subtelomeres are characteristically marked by the heterochromatic epigenetic mark H3K9me3 (Salcedo-Amaya *et al.* 2009). During transcription the active *var* gene is marked by more highly acetylated histone H4 and also H3K4me3 and H3K4me2 (Freitas-Junior *et al.* 2005; Lopez-Rubio *et al.* 2007). When not expressed it loses H3K4me3, but retains H3K4me2. This is thought to act as memory so that the same *var* gene is activated in the next invaded red cell (Lopez-Rubio *et al.* 2007). Conversely, methylation of H3K36 represses *var* gene expression (Jiang *et al.* 2013).

The mechanism for controlling monoallelic expression of *Babesia ves1* genes is less well understood. Expressed genes are found in a tandem configuration and are expressed from LATs by a bidirectional promoter. It is thought that chromatin remodelling is required for transcription suggesting there may be some commonalities with control of *var* expression (Huang *et al.* 2013). However, it is not clear that chromatin remodelling plays a direct role in monoallelic expression.

For all other apicomplexan families multiple members appear to be expressed at once. A small number of *P. knowlesi SICAvars* are expressed by the parasite at the same time (Barnwell *et al.* 1983). Interestingly none are expressed in splenectomized monkeys, suggesting that they are regulated in response to the host immune system (Barnwell *et al.* 1983). Such a response has also been observed for *pir* genes in several *Plasmodium* species. In *P. chabaudi* it was shown that parasites, which are serially passaged between mice, avoiding the mosquito and liver stages, express a reduced set of *pirs* and *RMP-fam-as* and that this is associated with an increase in virulence (Spence *et al.* 2013). Whereas perhaps 10–20% of the *pir* repertoire (20–40 genes) is expressed in serially blood-passaged parasites (Lawton *et al.* 2012), mosquito transmitted parasites express more than half of their repertoire (>100 genes), quite the opposite of what we would expect from genes involved in antigenic variation *sensu stricto* (Spence *et al.* 2013). Similar results have been shown for *Plasmodium yoelii*, with B or T cell deficient mice showing differences in expression of these genes (Cunningham *et al.* 2005).

Change in expression of the *srs* family of surface antigens in *T. gondii* is known to be important for evading the host immune response (Tomavo, 1996). Around half its repertoire of *srs* genes is expressed during the rapidly growing tachyzoite stage and multiple protein products have been localized to the parasite cell surface (Tomavo, 2001; Reid *et al.* 2012). During switch to the encysted, bradyzoite form, there is a switch to a largely non-overlapping set of *srs* genes (Jung *et al.* 2004). The regulatory mechanism controlling this is unknown.

Several families are known to localize within the host cell and have been shown to adapt the host cell to function as a better home for the parasite. *Fikk* kinases are expressed in the blood stage of *P. falciparum*, with different members expressed at different stages of development. Their products have been shown to locate to within the parasite itself, the Maurer's cleft in the host erythrocyte and to the inner side of the erythrocyte membrane (Nunes *et al.* 2007). Transcriptional data indicates that unlike *SfiI sub-telomeric* genes, *SVSP* family genes of *T. annulata* are expressed in a stage-specific manner by the macroschizont and that the majority of the gene products contribute to the secretome (Weir *et al.* 2010). These are predicted to localize to the host cytosol. No members of large gene families in *Theileria* are thought to localize to the parasite or host membranes.

Genomic data can give us clues about whether gene products are secreted, exported, membrane-bound or GPI-anchored. However, experimental approaches are required to confirm this and provide greater detail. There is great difficulty in localizing multiple members of protein families due to the requirement for specific antibodies or genetic manipulation of multiple, similar, genes. Improvements in locating the product of each family member over time would allow greater insight into a family's function.

## FUNCTIONAL ROLES

It is generally assumed that large variant gene families have a role in antigenic variation, this being the most straightforward explanation for maintaining such a large repertoire of related genes. However, it is clear that most large gene families in *Apicomplexa* are not involved in antigenic variation as it is often understood and many may not be involved in antigenic variation at all (Table 1). It has been proposed that gene families, which are involved in antigenic variation, might have a distinct primary function. The primary functions of *vars* for instance appear to be sequestration to avoid clearance by the spleen and rosetting to enhance efficiency of invasion (Rowe *et al.* 1997). They may vary greatly because they are exposed to the host immune system, not exposed to the host immune system simply in order to provide a variety of epitopes. Other *Plasmodium* species do not have *var* genes, or a family, which might obviously replace them, except perhaps for *SICAvar* in *P. knowlesi*. However *P. chabaudi* does sequester (Brugat *et al.* 2014) and shows antigenic variation (Brannan *et al.* 1994; Phillips *et al.* 1997), suggesting these functions can be achieved by genes with very different properties. It is possible that immune evasion could be mediated by expression of all or most of the repertoire of a gene family. Many similar proteins, expressed at relatively low levels, might prevent induction of an effective immune response, resulting in immune evasion without mutually exclusive expression (Cunningham *et al.* 2005, 2009). *Ves1* genes in *Babesia bovis* are implicated in antigenic variation and cytoadherence and, like *Plasmodium*, this species can sequester in the brain and can cause neurological damage (Sondgeroth *et al.* 2013).

*Pir* is the only large gene family conserved across all Plasmodium species examined. It has been shown that *pir* and *RMP-fam-a* expression (Table 1) is modulated in response to the host immune system (Cunningham *et al.* 2005; Spence *et al.* 2013). In particular, expression of a smaller repertoire of *P. chabaudi pirs* is associated with higher parasitemia and more damage to the host. These genes may have a role in attracting the immune system to a sufficient degree that it is prevented from doing excessive harm to the host.

A-type RIFINs and STEVORs have been shown to locate to the apical end of the invading merozoite stage of *P. falciparum*. It has been suggested that they provide a shield from immune attack against the more conserved proteins involved in invasion, through binding to erythrocyte surface molecules (Petter *et al.* 2007; Blythe *et al.* 2008). There is evidence for molecular mimicry amongst the related *pir* family in *P. knowlesi* (Pain *et al.* 2008). Seven *P. knowlesi pirs* contain regions of up to 36 amino acids, which perfectly match the immune system protein CD99 from the natural host *Macaca mulatta*. This would suggest that these *pirs* may compete with host CD99 for binding and could interfere with the host immune response.

Within large variant gene families individual members are able to mutate quite freely due to redundancy of function and reduced selective pressure. They may therefore have a great deal of opportunity to develop new functions. Despite the great differences in *var* gene repertoire, even between different strains of *P. falciparum*, a small number are have been under strong positive selection to maintain their structure and function (Rowe *et al.* 2002). *Var2csa* is able to bind chondroitin sulphate A on the placenta and its expression is associated with placental malaria in women pregnant for the first time (Salanti *et al.* 2004).

DBL*α* domains are found in *var* genes and in genes involved in invasion in *P. falciparum*. The *srs* gene family takes a similar multi-functional role in *Toxoplasma*. Host cell invasion by apicomplexans is preceded by attachment and reorientation (Carruthers and Boothroyd, 2007). In *Toxoplasma*, the same gene family involved in immune evasion is also involved in the first of these processes (Jung *et al.* 2004). SRS57 has been shown to act in binding host cells (Dzierszinski *et al.* 2000), while SRS34A has a role in attracting an immune response. SRS29B, SRS34A and SRS29C are specific to the rapidly growing tachyzoite stage and are strongly antigenic (Lekutis *et al.* 2001). Along with many other *srs* genes these are turned off during the switch to bradyzoites. The tachyzoite may attract the immune system, or the immune system may prompt the parasite to switch forms (Bohne *et al.* 1993). This may have a role in limiting parasite proliferation and also distracting attention from the bradyzoite, which can then hide in tissue and await transmission. Perhaps this family might have started out with a cell adhesion function, which showed antigenic variation because it was exposed. It could then have evolved a function in immune evasion switching which allowed the quiescent form to hide in tissues.

The intracellular stage of the bovine parasite *Theileria*, equivalent to the malaria blood stage, is not known to express any molecules on the surface of the invaded cell, a leucocyte (Boulter and Hall, 1999). The largest gene family in *Theileria, Tar/Tpr*, is predicted to encode mostly integral membrane proteins; however, no known function has been attributed to it (Weir *et al.* 2010). The next two largest variant gene families of *Theileria* appear to encode largely secreted proteins (Pain *et al.* 2005). It has been proposed that SVSPs have a role in transforming the host cell. They possess nuclear localization signals and it is thought they might localize to the host nucleus much like products of the smaller *TashAT* family (Shiels *et al.* 2004). Alternatively they might provide an immunological smokescreen effect by distracting immune cells from more conserved surface-bound antigens (Weir *et al.* 2010).

There are large kinase gene families from *Plasmodium* and *Coccidia*, which are also thought to be involved in modifying the host cell. *Plasmodium falciparum* has a unique expansion of the Apicomplexan-specific FIKK family of kinases (Nunes *et al.* 2007). There is evidence that some members are involved in phosphorylation of host membrane skeleton proteins and alter the mechanical properties of the red blood cell (Nunes *et al.* 2010). ROP kinases and inactivated pseudokinases are stored in club-shaped organelles known as rhoptries, which introduce their cargo to the host cell upon invasion. In *T. gondii*, ROP16 localizes to host nucleus where it activates the JAK-STAT pathway and modulates host gene expression (Saeij *et al.* 2007). TgROP18 localizes to the PVM (Taylor *et al.* 2006) and is able to prevent immune-related GTPases (IRGs) from attacking this membrane and destroying the parasite (Fentress *et al.* 2010). This family seems to provide a set of exquisite tools for modifying the host cell.

It has been proposed that *Cryptosporidium* species do not display any form of antigenic variation (Singh *et al.* 2005). Interestingly, they also have a paucity of large variant gene families. Only the mucin-like glycoproteins show a large expansion. These have been proposed to function in tethering the sporozoite stage to the oocyst wall. However, it is not clear why 30 genes would be required to perform this task. Why *Cryptosporidium* should be able to survive without the wealth of large gene families present in other apicomplexans is currently unclear.

Thus, a variety of roles have already been uncovered for large apicomplexan gene families. Most families however have no known function and await characterization (Table 1). Small research communities are assembling around some of these families, but many remain essentially unstudied.

## CONCLUSIONS

The *P. falciparum var* gene family is the most intensely studied large gene family in the *Apicomplexa*. It is involved in the best-understood mechanisms of antigenic variation and sequestration in this phylum. While other species exhibit antigenic variation and sequestration they use gene families with different properties and evolutionary histories. Many gene families are concerned with manipulating the host immune response or modifying the host cell rather than antigenic variation or sequestration. This review has highlighted some of the common themes and important contrasts that exist amongst large variant gene families in apicomplexan species. These may be useful in directing research to understand how apicomplexans interact with their hosts.
